# Remote sensing data and machine learning models estimate sorghum grain yield in a plant breeding program

**DOI:** 10.1016/j.plaphe.2026.100179

**Published:** 2026-02-05

**Authors:** N. Ace Pugh, Andrew Young, Matthew Nesbitt, Chad Hayes

**Affiliations:** Cropping Systems Research Laboratory, Plant Stress and Germplasm Development, USDA-ARS, Lubbock, TX, USA

**Keywords:** UAS, Plant breeding, Machine learning, Remote sensing, Sorghum, Deep learning, Yield

## Abstract

**P**henotyping remains a critical bottleneck in sorghum (*Sorghum bicolor* L. Moench) breeding programs, limiting rates of genetic gain due to labor-intensive yield estimation methods. To address this concern, this study investigates the potential of integrating remote sensing data with machine learning (ML) and deep learning (DL) models to improve sorghum grain yield predictions. Unmanned aircraft systems (UAS)-based imagery was collected across multiple field trials, extracting standard vegetation indices, canopy height features, and panicle traits using a YOLOv11-based object detection model, “YOLO-SORG.” Six ML models—including ridge regression (RR), elastic net (EN), LASSO regression (LR), support vector regression (SVR), random forest (RF), and XGBoost (XGB)—were trained to predict plot-level yield using three distinct feature sets: panicle traits, canopy traits, and a combination of both. Results indicate that models relying solely or partially on canopy-derived features provided the most consistent and accurate yield estimates (R^2^ ≈ 0.74–0.76), whereas models relying solely on panicle traits performed poorly (R^2^ ≈ 0.28–0.42), indicating nadir-derived panicle metrics were potentially being indirectly captured with the canopy traits. Traditional regression models outperformed tree-based ensemble methods in variance partitioning and repeatability (*R* ≈ 0.59-0.60), making them more suitable for many breeding applications. These findings highlight the promise of UAS-driven ML pipelines for non-destructive yield prediction but underscore potential limitations of nadir imagery for capturing panicle morphology and use in a robust yield prediction model. Future research should explore the inclusion of multi-temporal imaging, refined feature extraction approaches, and use of oblique, non-nadir imagery to enhance predictive accuracy in sorghum breeding programs.

## 1 Introduction

Sorghum (*Sorghum bicolor* L. Moench) holds a prominent position in global agriculture, ranking as the fifth most important cereal crop after maize, rice, wheat, and barley [1,2]. Its importance is particularly pronounced in arid and semi-arid regions of Asia and Africa, where its inherent resilience to drought and heat stress makes it a vital source of food. In the United States, sorghum is a critical crop for livestock feed and is gaining increasing favor with niche consumers of specialty products, such as gluten-free bread [3,4]. Sorghum breeders rely on precise and accurate yield data to select superior genotypes and accelerate the rate of genetic gain in their programs. At the same time, agronomists utilize yield estimates to optimize management practices and resource allocation [5,6]. Conventional methods for yield estimation in sorghum often involve manual sampling techniques, such as counting and weighing panicles from representative plants within a plot by hand or using a combine harvester to quickly gather data on entire plots [7]. While these methods can provide reasonably accurate estimates at small and large scales, respectively, they have several limitations. Sampling by hand or using machinery is inherently labor-intensive, expensive, and time-consuming, particularly in large fields or breeding trials with a large number of genotypes, which results in a phenotyping bottleneck for genetic improvement programs [8,9]. The limitations of traditional yield estimation methods underscore the need for efficient, non-destructive approaches that can provide accurate and timely yield predictions [10,11]. Machine and DL models using features derived from imagery captured by unmanned aircraft systems, or UAS, may help to address this need.

Unmanned aircraft systems, often called “drones,” equipped with high-resolution sensors have aided data acquisition in agriculture, offering unprecedented detail and efficiency for remote sensing applications [12,13]. In recent years, UAS-based remote sensing has been increasingly employed for yield estimation in various crops, including sorghum. Previous studies have demonstrated the potential of UAS-derived data for predicting sorghum yield [14,15]. These studies primarily focused on analyzing spectral vegetation indices, such as excess green index (ExG) and the Normalized Difference Vegetation Index (NDVI), and canopy height models derived from UAS imagery [14]. These indices provide valuable information about crop growth and biomass accumulation, which can be correlated with grain yield [16]. However, relying solely on these traditional features may not fully capture the complexity of yield formation in sorghum, which is a product of spatial and temporal factors such as soil, water, and pest presence [[17], [18], [19]]. In addition, potential morphological features such as panicle number, mean panicle size, and other potential yield determinants, such as photoperiod response, may not be directly captured by vegetation indices or canopy height that are extracted from nadir imagery [20]. Therefore, there is a need to explore more specialized approaches that can extract richer information from UAS data. To that end, ML and DL models may allow plant breeders and scientists to estimate grain yield in sorghum plots more efficiently and consistently.

Machine learning algorithms have emerged as a powerful tool for enhancing the accuracy and efficiency of yield prediction in agriculture, offering a data-driven approach to complement traditional methods [21,22]. Unlike traditional statistical approaches that rely on linear relationships and simplified assumptions, ML models can learn complex or non-linear patterns and interactions between predictor variables and yield [23]. This ability to capture intricate relationships is particularly valuable in agricultural systems where many interacting factors, including environmental conditions, management practices, and genetic traits can influence yield. By leveraging large datasets and sophisticated algorithms, ML models can identify subtle patterns and trends that conventional methods may miss. This enables more accurate and robust predictions, even in the presence of noisy data. Models that use regularization techniques such as RR, LR, and EN regression form the cornerstone of modern predictive modeling, addressing high-dimensionality and multicollinearity in complex datasets. Ridge regression introduces an L2 penalty that shrinks coefficient estimates towards zero, stabilizing estimates while retaining all predictors; LR, with its L1 penalty, promotes sparsity by effectively nullifying less informative variables, increasing model interpretability [24,25]. Elastic net combines these penalties, balancing the continuous shrinkage of RR with the feature selection benefits of LR, and is especially effective in scenarios involving correlated predictors [26]. Among the various ML algorithms, Random Forest (RF) and Extreme Gradient Boosting, or XGBoost (XGB), have demonstrated success in yield prediction across different crops [[27], [28], [29]]. These ensemble learning methods combine multiple decision trees to create a robust and accurate predictive model. They excel at handling high-dimensional data, such as that obtained from UAS-based remote sensing, and can effectively capture non-linear relationships between predictor variables and yield.

Deep learning (DL) models, such as convolutional neural networks (CNNs), have sparked a revolution in the computer vision space in recent years. They have demonstrated success in tasks such as object detection and image segmentation [30,31]. These models are designed to use visual data, mimicking the hierarchical structure of the human visual system to learn intricate patterns and features from images. This has led to significant advancements in various fields, including agriculture, where DL models are increasingly used for crop identification, disease detection, and yield estimation [30,32]. Among the various DL architectures, the “You Only Look Once” (YOLO) framework has emerged as a leading approach for object detection, renowned for its reported speed and accuracy [33,34]. Unlike traditional object detection methods that involve multiple stages, YOLO performs detection in a single pass, making it significantly faster and more efficient. This speed advantage is crucial for real-time applications and large-scale image analysis, which are common in agricultural settings. The YOLO framework has been successfully applied in various agricultural scenarios, including crop detection, disease identification, and weed mapping [[35], [36], [37]]. Its ability to accurately identify and locate objects within complex agricultural scenes makes it a valuable tool for automating tasks that traditionally require human expertise or labor. In the context of sorghum yield estimation, the YOLO framework holds potential for extracting valuable features that can enhance the accuracy of prediction models for key traits. For example, researchers can efficiently obtain precise panicle count information by training a YOLO model to identify and quantify individual panicles within UAS imagery. These features may directly relate to grain yield and can even potentially be integrated with traditional ML models to improve their predictive capabilities. Integrating DL-based feature extraction with traditional ML models offers a promising avenue for developing more accurate and robust yield prediction systems in sorghum, but it needs to be rigorously evaluated.

If the rate of genetic gain in sorghum breeding programs is to increase, breeders must be able to screen their material efficiently and precisely. This study investigates whether ML, DL, and combined models can be used to estimate grain yield in advanced sorghum trials. The specific objectives of this study were i) to develop a DL model to identify sorghum panicles in UAS imagery, named “YOLO-SORG,” based upon the YOLOv11 framework to estimate plot-wise sorghum panicle counts; ii) to test the traditional regression ML models for their ability to estimate yield using standard crop canopy features such as canopy cover, vegetation indices, and canopy height, with and without the DL-derived panicle traits included; and iii) to evaluate the ML models for their ability to be implemented within a hypothetical sorghum breeding program by estimating repeatability and selection capability.

## 2 Materials and methods

### 2.1 Germplasm and experimental design

**Fig. 1:**
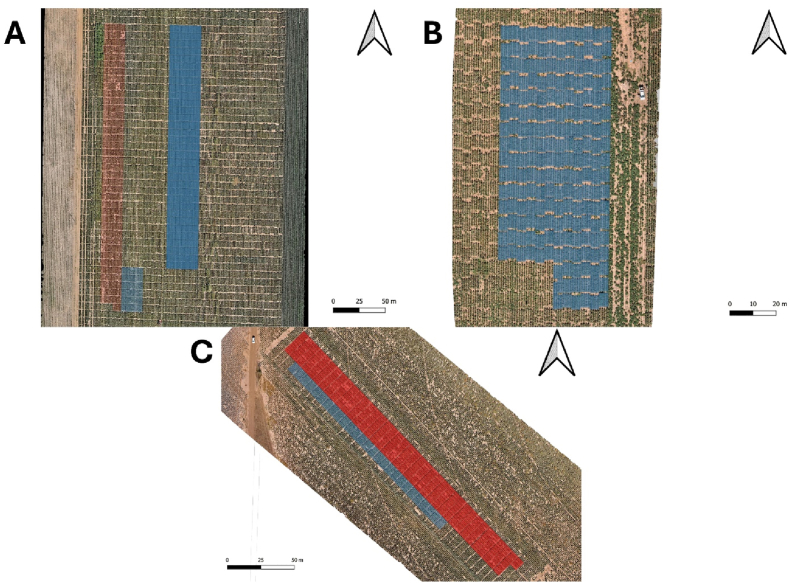
**Maps of Sorghum Breeding Field Experiments.** These maps show the three fields and their plots used for the training data in this study. The experiments were in Lubbock, TX in 2024 (A), Lubbock, TX in 2023 (B), and Abernathy, TX in 2024 (C). In addition, the AFTC experiment (in red) was in Lubbock and Abernathy in 2024 and used for a breeding analysis of the machine learning models used in the study.

A total of approximately 1820 two-row research plots were established across three sites in 2023 and 2024: USDA-ARS Abernathy 2024, Lubbock 2023, and Lubbock 2024 (Fig. 1).

Each plot consisted of two 20-ft, or 6.09-m rows spaced 40 inches, or 1.02-m apart and was sown with approximately 160 seeds to target a stand density of ∼50,000 plants per acre, or ∼123,555 plants per hectare. Border rows and alleys, or spaces between the plots, were excluded for downstream analyses. All germplasm comprised experimental grain sorghum hybrids managed by the USDA-ARS Lubbock breeding program; entries were designated as preliminary (first year of testing) or advanced (two or more years of evaluation), with preliminary hybrids generated by crossing novel inbreds to a common, elite tester line. The experimental design used was a randomized complete block design (RCBD) with four replications.

At the Lubbock 2023 and 2024 locations, irrigation was delivered via subsurface drip tape, whereas Abernathy plots received water from a center-pivoted system. Both sites averaged approximately 1.27 cm of applied water per week throughout the growing season. Before planting, every seed lot was treated with a safener, Concep III, to ensure uniform emergence and protect against herbicides. Planting took place on June 7, 2023 (Lubbock 2023), May 28, 2024 (Lubbock 2024), and June 17, 2024 (Abernathy). Mechanical harvesting was performed once grain moisture fell below 15%, using a Kincaid 8XP research combine retrofitted specifically for sorghum. Fresh plot grain weights were recorded in pounds (lb) and then converted to kilograms (kg) to derive final yield estimates.

### 2.2 UAS flight missions to collect remote sensing data

Approximately one to two weeks before harvest, UAS flight missions were executed across all research experiments. The surveys were performed using an Autel Evo II V3 Dual 640T (Autel Robotics Co., Ltd.) UAS equipped with a 50-megapixel RGB camera, ensuring high-resolution imagery for analysis. Flight parameters were optimized for each location: Lubbock 2023 flights were conducted at 20 m above ground level (AGL) with 80% front and side overlap, while both Lubbock 2024 and Abernathy 2024 employed parameters of 82% front and side overlap at 35 m AGL, yielding average ground sampling distances (GSD) of 0.6 cm, 1.1 cm, and 1.2 cm, respectively. A standard boustrophedon (i.e., back-and-forth) coverage pattern was used to ensure comprehensive and efficient spatial coverage. To enable precise georeferencing of the imagery, six ground control points (GCPs) were installed at each site, with one placed at each corner and two within the central area. This ensured the four to five GCP minimum required for robust georeferencing would still be visible by the end of the growth period. Global Positioning System (GPS) coordinates were collected for each GCP using a pair of Emlid RS2+ (Emlid Co.) devices, with one serving as a base point and the other used as a rover that could collect real-time kinematic positioning (RTK) data.

### 2.3 Photogrammetry, feature extraction, and Vegetation Index calculation

Photogrammetric processing of the UAS imagery was conducted using WebODM software (versions 3.5.3 and 3.5.5; https://github.com/OpenDroneMap/WebODM/) to generate orthophotos and DSMs. Lubbock 2023, captured on December 10, 2023, covered 0.012 km^2^ with 613 images and achieved an average ground sampling distance (GSD) of 0.6 cm; sparse reconstruction yielded a 99.9%-point recovery rate, and dense reconstruction produced approximately 61 million points, with GPS errors averaging 1.61 m and horizontal (CE90) and vertical (LE90) accuracies of 0.482 m and 3.473 m, respectively. Lubbock 2024, captured on September 26, 2024, spanned 0.090 km^2^ with 1404 images and an average GSD of 1.1 cm, resulting in a dense reconstruction of roughly 185 million points. Although the average GPS error reached 7.05 m, the implementation of GCPs reduced positional errors to near-zero, yielding horizontal and vertical relative accuracies of 0.061 m and 0.109 m, respectively. Finally, Abernathy 2024, captured on October 22, 2024, covered 0.069 km^2^ with 421 images and an average GSD of 1.2 cm, resulting in a dense reconstruction of approximately 58 million points, with average GPS errors of 1.41 m and relative horizontal and vertical accuracies of 0.066 m and 0.117 m, respectively, while GCP errors remained negligible. All image processing and statistical computations were implemented using a suite of Python libraries, including OpenCV (v4.5.3), NumPy (v1.21), pandas (v1.3.2), SciPy (v1.7.1), scikit-image (v0.18.3), and scikit-learn (v0.24.2). The workstation used to perform these processing steps was equipped with 32 CPUs and an NVIDIA RTX 2080 GPU. The dataset comprised standard red-green-blue (RGB) images collected via UAS. All images were systematically organized, with their file paths cataloged in a master CSV file to enable precise mapping between corresponding RGB and digital surface map (DSM) data for subsequent analyses. Red-green-blue image processing commenced by calculating key statistical features for each color channel including the mean, standard deviation, skewness, kurtosis, and entropy. In addition, a suite of vegetation indices pertinent to crop health and canopy characteristics was derived using established pixel-level arithmetic formulas. These indices included Excess Green (ExG), Excess Red (ExR), ExGR, the Color Index of Vegetation Extraction (CIVE), the Vegetative (VEG) Index, the Green Leaf Index (GLI), the Visible Atmospherically Resistant Index (VARI), the Triangular Greenness Index (TGI), the Normalized Green-Red Difference Index (NGRDI), the Normalized Green-Blue Difference Index (NGBDI), the Red-Green Ratio Index (RGRI), the Green-Blue Ratio Index (GBRI), the RGB-based Vegetation Index (RGBVI), and the Modified Green-Red Vegetation Index. Plot-level mean values for each derived metric were subsequently recorded to characterize the canopy structure effectively.

For digital surface map (DSM) image processing, only pixels corresponding to valid elevation ranges (specifically between 937 and 990 m above sea level (ASL) as determined by site-specific criteria) were retained. To extract canopy height, the elevation of the surrounding soil pixels within each extracted plot image was subtracted from the elevation of the canopy pixels. This method was chosen because of a lack of early flights where the ground was bare, which are required to do a common alternative where canopy height maps (CHM) are calculated by subtracting a DSM from a digital terrain map (DTM). For the extracted groups of pixels, summary statistics for height, such as mean, standard deviation, variance, minimum, maximum, and standard error were computed alongside a range of percentiles (from the 1st to the 100th percentile), ensuring a comprehensive statistical description of elevation data. Furthermore, texture analysis was performed on both RGB and DSM images to quantify surface roughness and structural variation. Red-green-blue images were first converted to grayscale, and the grey-level co-occurrence matrix (GLCM) method was employed to extract texture metrics such as contrast, correlation, energy, and homogeneity. For DSM images, gradient-based metrics like terrain roughness (slope) were calculated using numerical gradient techniques, and Histogram of Oriented Gradients (HOG) features were extracted to further characterize structural patterns relevant for crop modeling and terrain analysis.

Given the scale of the dataset, parallel computing techniques were used for efficient processing. Python's module enabled each RGB-DSM image pair to be processed independently across multiple CPU cores, significantly reducing overall computation time. All computed statistical features were stored in structured CSV files, after which Z-score normalization was applied. This normalization step ensured consistency and comparability across the dataset for subsequent ML analyses.

### 2.4 Training and testing of YOLO-SORG deep learning model

The panicle detection model was developed using the YOLOv11-Medium framework, a state-of-the-art single-pass object detection system that balances speed and accuracy [38,39]. YOLO, an acronym for “You Only Look Once,” has been continuously developed, with YOLOv11 incorporating architectural improvements such as the C3k2 block and C2PSA module, which significantly enhance detection performance [40]. This mid-sized, pre-trained variant is ideally suited for high-throughput applications, such as analyzing UAS imagery, and was implemented on a workstation equipped with 32 CPUs and an NVIDIA RTX 2080 GPU. The computational environment comprised Python 3.9, CUDA 12.4, PyTorch 2.5.1, and OpenCV 4.10.0 [41,42].

**Fig. 2:**
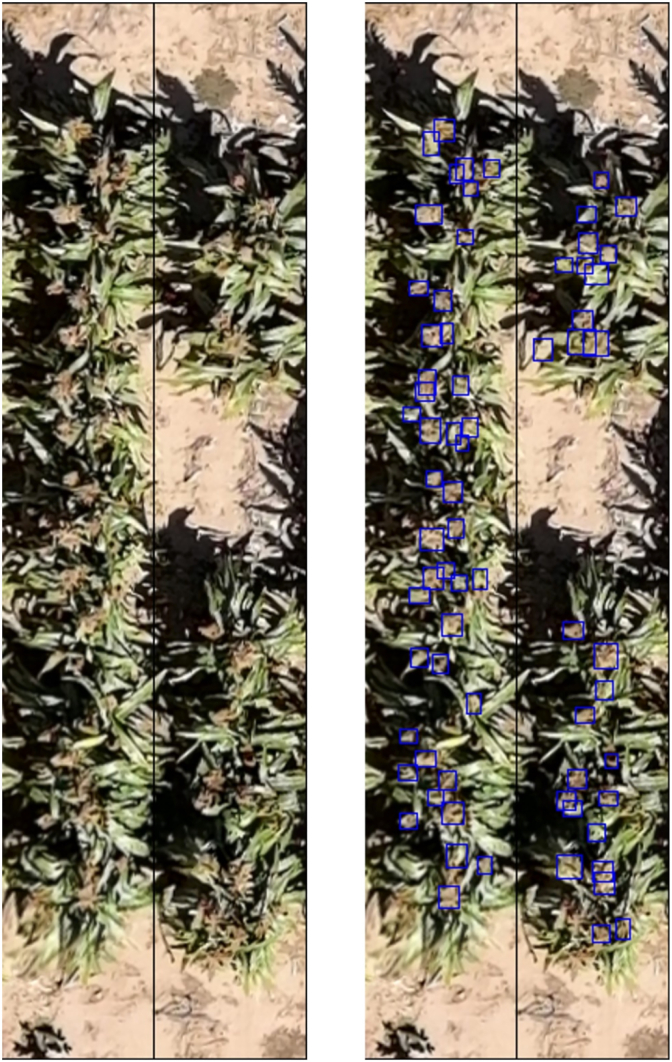
**Demonstration of the YOLO-SORG model for detection of panicles.** This representative plot (left panel) shows how the detection algorithm identifies and counts the panicles (right panel). Note that despite significant amounts of training data, the algorithm still makes misidentifications, particularly when panicles have lighter seed colors.

The model—referred to hereafter as the YOLO-SORG model—was trained using 1119 RGB images of sorghum containing visible panicles (Fig. 2). High-resolution nadir UAS and ground-based images were segmented into 640 × 640-pixel sub-images to facilitate model training. Approximately 25,000 individual panicles were manually annotated using Roboflow's online annotation tool, providing a robust ground truth for subsequent training [43,44]. To enhance model generalizability, an array of data augmentation techniques were applied, including multi-scale resizing (ranging from 50% to 150% of the original image dimensions), horizontal flipping, shifts in the HSV color space, mild blurring and erosion, mosaic augmentation (combining four images into one), and AutoAugment with RandAugment.

Training was conducted over 300 epochs with the following settings: an input image size of 640 × 640 pixels, a batch size of 24, and an initial and final learning rate of 0.01. The training further employed a momentum of 0.937, a weight decay of 0.0005, and a three-epoch warmup period during which momentum was ramped from 0.8. Mixed precision training was enabled, and loss weights were set to 7.5 for box loss, 0.5 for class loss, and 1.5 for DFL (Distribution Focal Loss), with an intersection-over-union (IoU) threshold of 0.7 for non-maximum suppression (NMS) and dropout fixed at 0.0. The 0.7 NMS threshold was chosen so that features identified were less likely to be extraneous, and more likely to be features valuable for panicle detection. In addition, this threshold would help to avoid duplications where too many bounding boxes were created for overlapping or adjacent panicles. Two checkpoints, last. pt and best. pt, were saved during training, with the latter being used for all downstream tasks. Notably, the model's performance, measured in terms of precision (68.2%), recall (63.1%), and detection stability, steadily improved, with optimal results observed around epoch 86.

Post-training, the best. pt model was applied to UAS plot-level imagery acquired for this study. Inference was executed on both GPU and CPU platforms to ensure compatibility across different computational methods. The detection pipeline involved running the model with a confidence threshold of 0.25 and an NMS IoU threshold of 0.45, followed by size-based bounding box filtering, additional NMS, and extraction of key metrics such as per-image panicle count and average bounding box size. For testing the model in this fashion, only UAS nadir images were used. The resultant data were saved as annotated images and CSV summaries, and these features were further analyzed both in isolation and in combination with canopy characteristics to support subsequent traditional ML models.

### 2.5 Training and testing of machine learning models

A panel of several different ML models were tested for their ability to estimate plot yield in this study to ensure that an optimal model could be identified. The set of models tested included several linear regression models (RR, LR, and EN), support vector regression (SVR), and two ensemble models that use decision trees (RF and XGB). Each of the models was run in Python using 50 random states, or iterations, with a comprehensive grid search performed prior to each random state to automatically identify and use optimized hyperparameters [45,46]. Three different feature sets were used to model yield values, each of which contained various combinations of extracted features. First, we attempted to use a feature set (Panicle Traits) consisting only of the panicle count and bounding box data derived from the YOLO-SORG DL model. Second, we tested a feature set (Canopy Traits) that consisted entirely of ‘standard’ canopy estimates extracted from each field plot, such as canopy cover and excess green index (ExG). Finally, we also tested a feature set that was a combination of the panicle traits and the canopy traits (Combined). Data cleaning was performed prior to running the models to eliminate clear outliers or mistakes by the harvest monitor or combine. Data were Z-score normalized before running any of the models, but other transformations prior to model training were unnecessary [47]. The mean and standard deviation of the R^2^ and root mean square error (RMSE) values across all random states were saved for each model, as well as the ‘best’ random state and its associated yield estimates. The ‘best’ random state was determined to be the one that had the highest test R^2^ value. The overall best model obtained from the best random state was used for latter sections of this study to assess the value of these model estimates in a breeding program, described in the next section. The workstation used to train and test these models was equipped with 40 CPUs and an NVIDIA RTX A5000 GPU.

Ridge, LR, and EN regression methods were performed by calling ‘sklearn.linear_model’ within the Scikit-learn or ‘sklearn’ library in Python [48]. Each of these models uses different regularization techniques to handle collinear data, overfitting, and model instability via the addition of penalty terms to the loss function. Ridge regression, otherwise known as L2 regularization, introduces an L2 penalty, i.e., adding the sum of the squared coefficients to the loss function [24]. This uniformly shrinks the coefficient estimates and thus helps to stabilize the model, even though it retains all predictors. Least Absolute Shrinkage and Selection Operator (LASSO) regression on the other hand, incorporates an L1 penalty by adding the sum of the absolute coefficient values to the loss function [25]. This approach not only shrinks the coefficients but can also force some to become exactly zero, thereby performing an implicit variable selection that simplifies the model and enhances interpretability. Elastic Net regression combines both L1 and L2 penalties, offering a balanced compromise that is especially useful when predictors are correlated [26]. By tuning the contribution of each penalty via a mixing parameter, EN can selectively shrink coefficients while benefiting from both the variable-selection properties of LR and the stability provided by RR.

Support vector regression was performed using the ‘sklearn.svm’ library in Python. Support vector regression is a supervised learning model that extends the principles of SVM to regression problems by constructing a function that approximates the relationship between input features and continuous outputs with controlled error tolerance [49,50]. In SVR, the objective is to determine a regression function that deviates from the actual target values by no more than a pre-specified threshold, known as epsilon, while simultaneously ensuring the function remains as flat as possible. Errors within the epsilon-insensitive tube are disregarded, whereas deviations exceeding this margin incur a penalty, thereby focusing the model's attention on the most informative data points, termed support vectors. This formulation results in a convex optimization problem that achieves a balance between model complexity and predictive accuracy. Furthermore, through the application of kernel functions, SVR can effectively handle non-linear relationships by implicitly mapping the original input space to a higher-dimensional feature space, where a linear regression function is determined.

The RF model was tested by calling ‘sklearn.ensemble’ from the ‘sklearn’ library in Python and XGB was performed using the ‘xgboost’ library in Python [48,51,52]. As with the other models, these models were run across 50 random states or iterations, with an extensive grid search performed to optimize hyperparameters prior to running each random state. Random Forest and XGB represent two sophisticated ensemble learning techniques that have been extensively tested and implemented for both classification and regression tasks due to their robustness and predictive accuracy. Random Forest operates by constructing a multitude of decision trees during training, where each tree is built on a bootstrap sample of the data with a random subset of features considered at each split; this deliberate injection of randomness not only reduces overfitting by averaging the predictions from diverse trees but also enhances overall model stability and generalization [51]. In contrast, XGB uses a gradient boosting framework whereby trees are constructed sequentially to iteratively minimize a predefined loss function, using both first and second order derivatives to determine the optimal split; this process is further refined through regularization strategies that control model complexity and mitigate the risk of overfitting [52]. The application of these methods highlights the strengths of ensemble methodologies in capturing complex nonlinear relationships and achieving high levels of predictive performance. In addition, tree-based ensemble models can potentially arrive at estimates using more complex interactions between features than the linear regression models can [53].

For statistical evaluation of the ability for ML-derived yield estimates to be used in a breeding program, it was important to ensure that data leakage did not occur, since it would be impossible to use yield data collected by a combine to check ML predictions in a real-world scenario where these models were meant to be a substitution for manual harvesting. To that end, one breeding trial that was present in two environments, Lubbock and Abernathy in 2024, was identified. This trial, called AFTC, is an advanced grain sorghum performance trial evaluating the agronomic performance of experimental seed-parent (B-line) inbreds in combination with diverse pollinator (R-line) inbreds. The trial was left out of the training data, and the best model using the Canopy feature set was used for training on the rest of the plots. Using the ‘pickle’ library in Python, the best performing algorithm for each ML model was saved and used to make ‘blind’ predictions on the AFTC test in both locations, treating it as a test dataset to understand model generalizability. The yield estimates derived in this fashion were used for the rest of the analyses in this study, including estimation of variance components and repeatability.

### 2.6 Data analysis and statistics

Pearson's correlation coefficients (r) were calculated for each of the features extracted from the imagery along with the ground-truth, actual estimates of yield values in the study. The ground-truth yield values collected by the combine harvester as well as the predicted yield values derived from the ML models were used to conduct restricted maximum likelihood (REML) analysis across environments using Fit Model (all random) in JMP Pro software [54,55]. This analysis was conducted using the statistical model

$$Y=\alpha_{i}+\beta_{j}\left[\gamma_{k}\right]+\gamma_{k}+\left(\alpha_{i}*\gamma_{k}\right)+\varepsilon$$

where *Y* is the ground-truth or ML-derived estimate of grain yield, α is the genotype (*i*), $\beta_{j}\left[\gamma_{k}\right]$ is the replication β (*j*) nested within the environment γ (*k*), γ (*k*) is the environment, $\left(\alpha_{i}*\gamma_{k}\right)$ is the interaction between genotype and environment, and ε is the error. Because this method assumes that the distribution of the residuals is normal, and they were found to significantly differ from normality according to a Shapiro-Wilk test, it was considered necessary to perform a Box-Cox transformation on the data to achieve a distribution closer to normality and rerun the REML [56]. The results using the transformed data were the data used for subsequent analyses. The REML process provided information about the percentage of total variance that was explained by each of the components, which also allowed for the calculation of repeatability (*R*) estimates. Repeatability was calculated using the equation

$$R=\frac{\sigma_{g}^{2}}{\sigma_{g}^{2}+\frac{\sigma_{ge}^{2}}{e}+\frac{\sigma_{\epsilon }^{2}}{re}}$$

where *R* is the repeatability estimate, $\sigma_{g}^{2}$ is the genotypic variance, $\sigma_{ge}^{2}$ is the variance for the genotype × environment interaction term, $\sigma_{\varepsilon }^{2}$ is the error variance, r is the number of replications (2), and e is the number of environments (2) [57]. Repeatability is a metric like heritability (*H*^*2*^), which is frequently used by plant breeders and other crop improvement scientists to determine how consistent their methodologies are and how successful efforts to select phenotypic differences will be [58]. However, it is distinct from heritability because it does not assume that the population possesses a familial structure and does not directly give information about how a phenotype will be genetically improved in that population.

Once the ability for the ML models to capture genetic variation and their repeatability were evaluated, it was important to determine how well the ML estimates of yield might perform when making selections. Because REML had already been conducted for the analysis of variance explained, the best linear unbiased predictors (BLUP) were also calculated for each of the genotypes in the study using JMP Pro software [55]. As their name suggests, BLUPs are a robust method for estimating the relative performance of genotypes in a study while mitigating potential bias caused by differential performance across environments [59,60]. Using the variance components to arrive at a breeding value can help to reduce potential errors when making selections or ranking the performance of genotypes across multiple replications and/or environments. To determine if the ranking of genotypes was similar between the ground-truth data and the predicted yield estimates, the BLUPs were used to perform two sets of analyses. First, to examine the comparison of rankings between methods, Spearman's correlation coefficients (*r*) were calculated. This method allows for a comparison of how the relative ranking of entries compares between two methods, rather than using the value itself. Higher Spearman's *r* values indicate that the rankings of entries are more similar. Second, confusion matrix-style visualizations were used to gain a clearer understanding of how ML-generated yield values would perform for making selections [61]. Confusion matrices allow a researcher to determine how many entries or genotypes align to the same ‘bin’ in their data. Although confusion matrices are most often used for classification algorithms where discrete categories are present in the data, they can still be a helpful way to visualize the outputs of a regression model if the data can be separated into informative groups. To simulate typical breeding categories, four major bins were used: the top 10% yield, the top 25%, the top 50%, and the bottom 50%. The top 10% and 25% would generally be advanced within a program, while the bottom 50% would usually be eliminated. It is important to note that the bins are nested within the next highest bin; e.g. the top 50% also includes the top 25% and top 10%. Therefore, these categories should not be understood to be mutually exclusive, as is usually the case when using confusion matrices.

## 3 Results

### 3.1 Correlations between ground-truth yield estimates and features extracted from remote sensing data

**Fig. 3:**
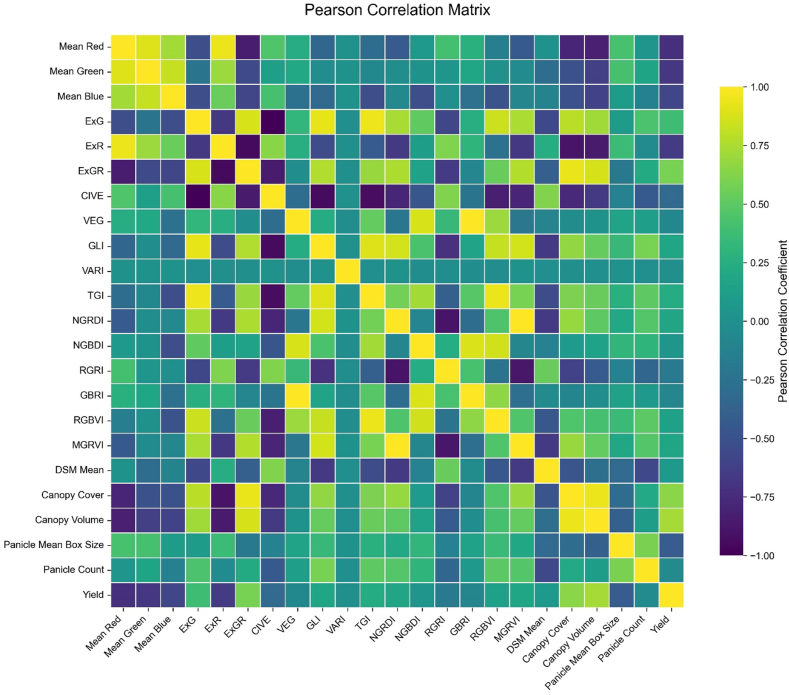
**Heatmap of Pearson's Correlation Coefficient Values for Features.** This heatmap shows the correlation (*r*) between a suite of remote sensing-derived features and grain yield in sorghum. Correlation significance is not indicated.

Correlations between the extracted features and grain yield varied considerably (Fig. 3). For instance, mean RGB values and ExR demonstrated moderate to high negative correlations with yield, whereas canopy cover and canopy volume exhibited strong positive correlations. In contrast, many of the other vegetation indices yielded only low to moderate correlations. Given that different indices may capture distinct aspects of an image, integrating multiple traits with similar correlation patterns into a complex ML model could enhance yield prediction. Importantly, no single feature assessed in this study showed a sufficiently strong correlation to serve as an independent predictor of yield, highlighting the usefulness of a more sophisticated ML model.

### 3.2 Performance of machine learning models for predicting plot-level yield estimates

**Fig. 4:**
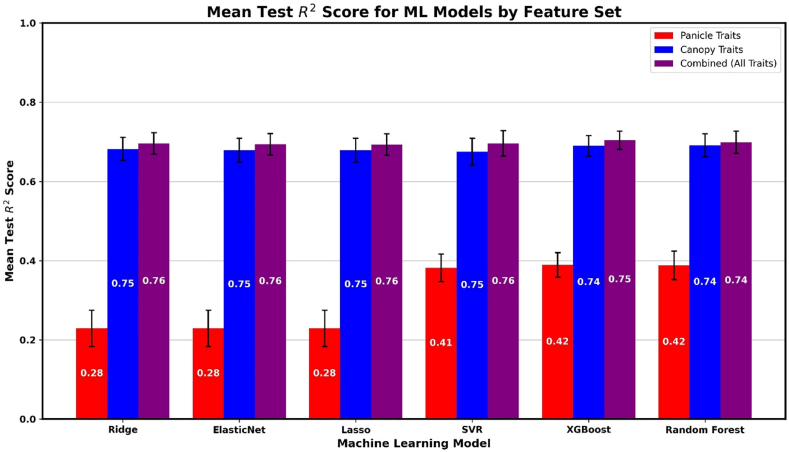
**Mean Test R^2^ Scores for Machine Learning Models by Feature Set.** This bar graph summarizes the performance of six machine learning models: ridge, elastic net, lasso, support vector regression (SVR), XGBoost, and random forest by displaying the mean and standard deviation of the R^2^ score over 50 iterations. Three feature sets were evaluated: Panicle Traits (Red), which includes only panicle counts and bounding box dimensions obtained using the YOLO-SORG deep learning model; Canopy Traits (Blue), which consists of features derived solely from the canopy (e.g., canopy height, canopy cover, vegetation indices); and Combined (All Traits, Purple), which integrates both the panicle and canopy traits. The value at the center of each bar represents the highest R^2^ achieved by each model based on the optimal random state, reflecting their peak performance.

**Fig. 5:**
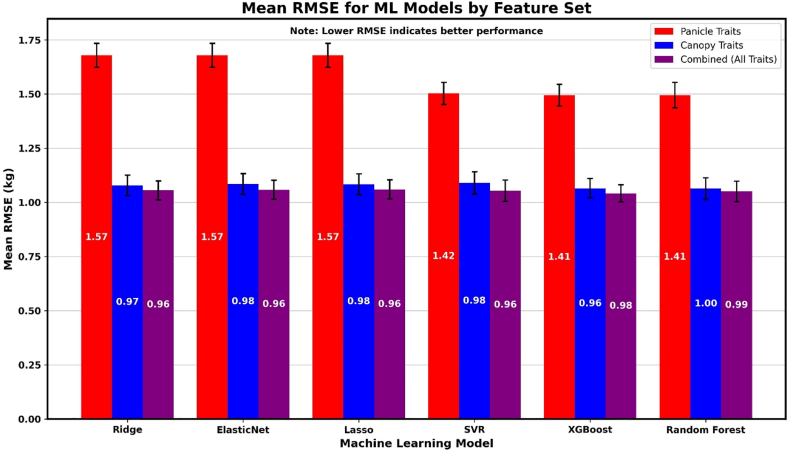
**Mean RMSE Values for Machine Learning Models by Feature Set.** This bar graph summarizes the performance of six machine learning models: ridge, elastic net, lasso, support vector regression (SVR), XGBoost, and random forest by displaying the mean and standard deviation of the RMSE score (kg) over 50 iterations. Three feature sets were evaluated: Panicle Traits (Red), which includes only panicle counts and bounding box dimensions obtained using the YOLO-SORG deep learning model; Canopy Traits (Blue), which consists of features derived solely from the canopy (e.g., canopy height, canopy cover, vegetation indices); and Combined (All Traits, Purple), which integrates both the panicle and canopy traits. The value at the center of each bar represents the lowest RMSE achieved by each model based on the optimal random state, reflecting their peak performance. Lower RMSE values correspond to superior predictive accuracy.

The performance of the six models was comparable when using the Canopy Traits and Combined feature sets (Fig. 4, Fig. 5). In contrast, the Panicle Traits feature set resulted in considerably poorer performance with the RR, EN, and LR models (R^2^ = 0.28; RMSE = 1.57 kg). Although performance improved somewhat for the SVR, RF, and XGB models with the Panicle features, test metrics remained suboptimal, with R^2^ values between 0.41 and 0.42 and RMSE ranging from 1.41 to 1.42 kg.

Notably, augmenting the Canopy Traits set with panicle traits did not yield improved performance over the Canopy Traits alone. The models based solely on canopy information exhibited R^2^ values of 0.74–0.75, while those built on the Combined Traits set ranged from 0.74 to 0.76. This was unexpected given that panicle count is frequently presented as a robust potential predictor of yields. Nonetheless, the strong performance of the canopy-based models was evident across all approaches. Furthermore, the RR, EN, and LR models produced marginally better results in their optimal configurations than did XGB and RF, indicating that the reduced efficacy was primarily linked to the use of panicle features in isolation.

### 3.3 Percentage of variance explained, and repeatability of predicted and ground-truth yield estimates on a genotypic basis

**Fig. 6:**
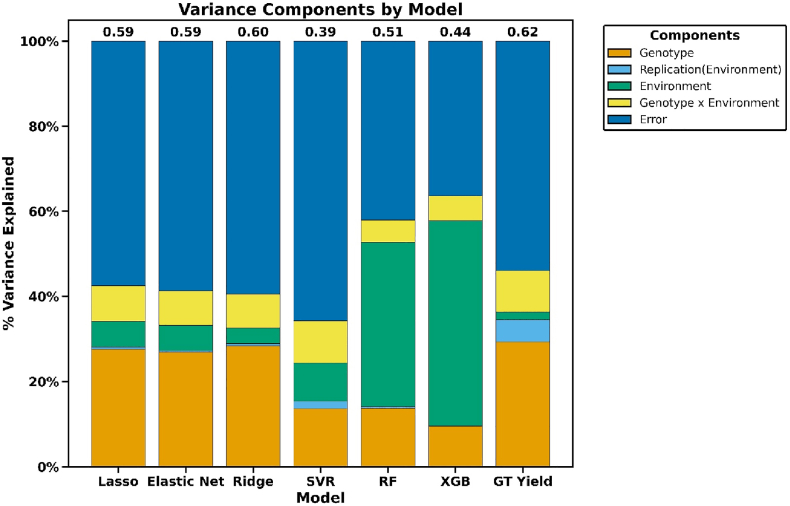
**Percentage of Variance Explained by Model Component and Repeatability Estimates.** This bar graph depicts the percentage of variance explained by the components of the mixed model: namely, Genotype, Replication nested within Environment, Environment, the Genotype × Environment interaction, and the Error term. Additionally, the repeatability (*R*) for each component is indicated at the top of each corresponding bar.

The percentage of variance explained revealed more pronounced differences among the ML models than did R^2^ or RMSE (Fig. 6). An analysis of the error partitioning across components indicated that the LR, RR, and EN models more closely mirrored the ground-truth (GT), combine harvester target data, in contrast to the SVR, RF, and XGB models. Random forest and XGB assigned a substantially higher proportion of variance to the Environment component, whereas the ground-truth model allocated only a modest fraction to this factor. Moreover, the genotype effect was markedly lower for these two models compared to both the other models and the ground-truth. As a result, the repeatability metrics for SVR (R = 0.39), RF (R = 0.51), and XGB (R = 0.44) were considerably lower than those observed for the LR, RR, and EN models, which ranged from R = 0.59 to 0.60. The RR model exhibited variance allocations and repeatability values closely aligned with the ground-truth data, suggesting that it partitions error in a manner likely preferable to many sorghum breeders and researchers due to general preference toward maximizing genotypic variance while minimizing the error variance.

### 3.4 Ranking of genotypes for each model and ability to be used to make selections in a sorghum breeding program

**Fig. 7:**
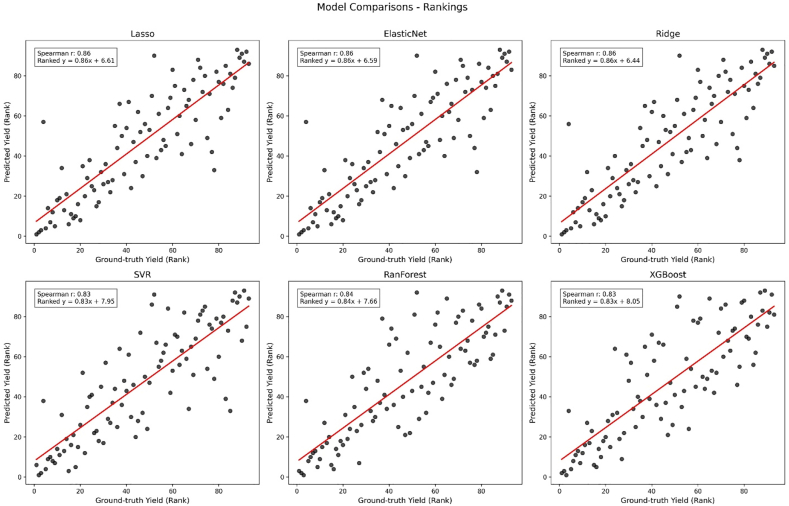
**Spearman's Rank Correlations for Yields Based on Entry BLUPs.** These graphs present the Spearman's rank correlation plots for each machine learning model examined in this study. Higher Spearman's coefficients (*r*) signify that the ordering of entries based on their best linear unbiased predictors, or BLUPs (breeding values computed using variance components) is more consistent across methods. Additionally, the regression equation for each red fit line is displayed. It is important to note that these rankings reflect only the similarity in order of the entry yields—not differences in the magnitude or range of values between the two datasets.

The Spearman's rank correlation coefficients for all six models were high (Fig. 7). While each model effectively ranked the genotypes based on BLUPs, the LR, RR, and EN models demonstrated better performance. This observation aligns with the variance explained and repeatability estimates (Fig. 6), where these three models closely approximated the ground-truth yield measurements obtained via combine harvester. When ranking performance was the sole criterion, however, all six models produced reasonable results. Importantly, the ranking performance is a visualization of the similarity in rank between the models but does not indicate that the actual values of the data are the same.

**Fig. 8:**
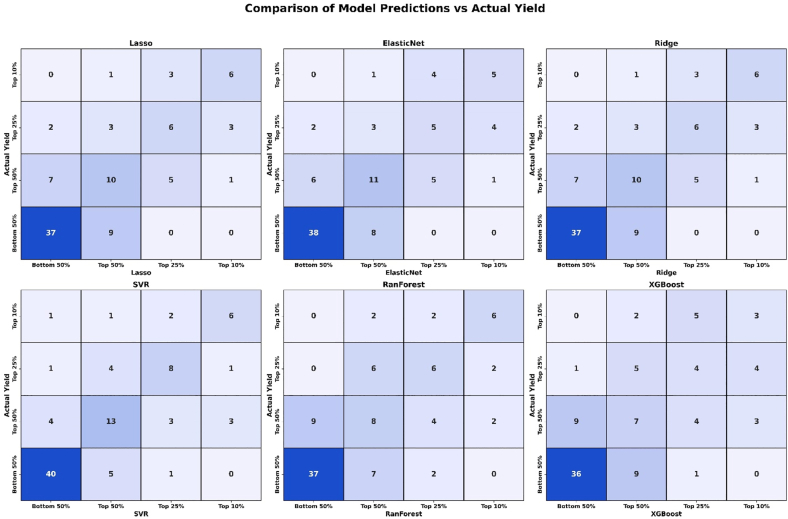
**Plant Breeder Ranking Comparison of Model Predictions vs Actual Yield.** These confusion matrix-style figures illustrate the accuracy with which the various ML-derived yield estimates could be sorted into predetermined yield-based bins relative to the ground-truth (Actual Yield) estimates. Four bins were defined: the bottom 50%—typically eliminated from a breeding program; the top 50%—retained for further evaluation; the top 25%—which are often selected but are not regarded as top performers; and the top 10%—generally earmarked for release or continuation. The Top 50%, 25%, and 10% categories are nested, not mutually exclusive. In these matrices, higher numerical values accompanied by bluer colors indicate that the entries within that category were more accurately sorted.

The confusion matrices reinforced these findings (Fig. 8). The LR, RR, and EN models maintained robust performance. In contrast, the SVR model committed a particularly consequential error by misclassifying an entry—one deemed to be in the lower 50% of the material was, in fact, among the top 10% of performers. Such a misclassification is critical, as it could result in the inadvertent exclusion of a high-performing genotype from a breeding program. Conversely, errors that led to a top 10% entry being assigned a modestly lower ranking were less detrimental, since the entry would still hypothetically fall within the top 50%. Overall, while the models excelled at eliminating the poorest performers, they were somewhat less reliable in identifying the very best.

## 4 Discussion

### 4.1 Performance of different ML models for estimating plot-level sorghum yield

This study sought to determine which model would perform the best for estimating yield, regardless of how complex the model might be. Interestingly, the simpler models (RR, EN, and LR) that incorporate regularization terms all performed comparably to the more complex decision tree-based models like RF and XGBoost when canopy traits were included in the feature set (Figs. 4 and 5). This result was unexpected since RF and XGB can often identify more complex relationships and arrive at better predictions at the cost of increased computational burden and time [62]. That was not the case in this study, where RR and the other simpler models still arrived at similar plot-level results. The exception to this was when running the Panicle Traits models, where the RF and XGB models performed much better. Regardless, that feature set's performance remained insufficient for implementation in a breeding program since it reached R^2^ scores of only 0.41-0.42 (Fig. 4). Once the canopy traits were added to the feature set, the performance of the various algorithms was much more comparable.

Despite this being an unexpected result in the current study, it is widely acknowledged that the more complex model is not necessarily always the best model for every task [62]. Even where RF or XGBoost perform slightly better at plot-level predictions, as in this study, it is always crucial to compare those perceived benefits to the additional computational cost that may be involved with using those models [63]. Although all models ran in a reasonable time frame on the GIS-capable workstation used to perform the modeling in this study, that may not be a realistic hardware expectation for many users, and further study should be conducted to compare benchmarks. In addition, the easiest models to integrate into an efficient workflow or to use for rapid in-field determinations are typically the ones that are the least computationally intensive [64]. As mentioned previously, the exception to this trend of simpler models performing the same as the decision tree-based models was when using Panicle Traits as the only features in the model (Figs. 4 and 5). In these cases, RF and XGB performed better than the RR, EN, or LR methods. This still did not lead to an acceptably accurate prediction model, but it did highlight the potential ability for RF and XGB to do ‘more with less’ as compared to RF, EN, and LR. Beyond this model performance difference, it was also unexpected that panicle number and panicle width did not lead to strong predictions of yield on their own.

Many recent studies explicitly or implicitly suggest that panicle count is a key component of yield, therefore a DL model that can count and estimate the number and bounding box size of panicles may allow for accurate and precise estimates of the trait [20,65,66]. That did not appear to be the case in this study, where the Panicle Traits feature set performed much worse than the Canopy Traits feature set and, additionally, did not offer any tangible benefit when evaluating the Combined Traits feature set (Figs. 4 and 5). There are several caveats to mention when discussing these results. First, it is important to note that the imagery collected by a UAS is typically taken and extracted at nadir (top-down), as it was in this study. This inevitably means that a user is limited to panicle counting and obtaining estimates of top-down panicle width, but there is no way to account for panicle length or how densely packed (open vs closed) the panicles are, which are likely to be critical morphological traits to consider when discussing yield estimation [67]. This limitation could potentially be addressed by using supplementary oblique flights over the material; however, this requires much longer flight missions and makes processing, extraction, and analysis of informative features much more complex [68,69]. Another caveat is that the determinations made in our study and its experimental material may not be applicable in all cases, so it will be important to further evaluate this finding in a diversity of populations and environments. For example, our population of genotypes was composed of advanced breeding material with limited variability between varieties. A more variable population, such as material earlier in a crop improvement pipeline that varies more widely for yield, could lead to better results. Therefore, this finding should not be understood to be prescriptive for all future potential applications for panicle identification and segmentation DL models. Even so, it does appear that canopy features such as mean RGB, texture differences, and others are somehow indirectly capturing the value of nadir-derived panicle data in this material without the need for a DL model to capture and segment them separately.

### 4.2 Evaluation of ML models for use in a plant breeding program

A variety of ML algorithms have been successfully tested and applied in prior studies across multiple crop species. Random forest and XGB are frequently effective but require caution due to potential overfitting and lack of inherent interpretability [28,29]. In Guo et al. a variety of models were tested. Hyperspectral data were used with a random forest algorithm to obtain a high prediction accuracy for maize yield at R^2^ = 0.92 [70]. In Yang et al. XGB was a top performer for estimating wheat yield using UAS data. [71]. Pugh et al. determined that XGB and RF performed similarly when multiple environments were included in the dataset, but performance within singular environments was variable [72]. Support Vector Machines, RR, EN, LR, and DL frameworks like YOLO are also commonly employed with varying degrees of success depending on the dataset and context [23,[73], [74], [75]]. Support vector regression has been used in several studies to estimate crop yields at scale using combinations of remote sensing and environmental features [76,77]. As mentioned, DL has served as a key emerging technology for estimating crop yields. For example, in Escalante et al. a DL model was used to extract features to estimate nitrogen fertilization levels as well as predict barley yields at 83% accuracy [78]. A DL model used by Sun et al. demonstrated that hyperspectral data can be used to estimate yield and lodging in soybean [79]. Despite these successes in other crops, it did not appear that DL-derived panicle counts and widths were useful for yield estimation in our study. It is critical to note that our study also did not evaluate the practice of ‘stacking’ multiple traditional ML models' results into an ensemble model. Ensemble learning methods that combine predictions from multiple base models (e.g., stacking, decision-level fusion) into a combined ‘metamodel’ sometimes demonstrate superior performance and robustness compared to individual ML algorithms, although great care must be taken to avoid data leakage during training [[80], [81], [82]]. This presents as a potential avenue to further evaluate ML for yield prediction in sorghum, although preliminary executions of the method we tested did not lead to any noticeable differences in performance.

An important consideration for our study is that multi-temporal data collected over the course of the growing period were not available for the experiments. Indeed, previous research has demonstrated that growth dynamics may provide further insight into the ultimate performance of a crop variety [83]. While optimal single time points for prediction often occur during mid-to-late reproductive stages (e.g., flowering to milk stage or grain filling), significant variability exists between genotypes, environments, and years. Critically, integrating data from multiple growth stages can lead to improvements in prediction accuracy and model robustness compared to single-date approaches, effectively capturing the cumulative impact of development processes on final yield [15,83,84]. It may be valuable to collect several important timepoints (e.g., peak vegetative stage, flowering, etc.) and incorporate them into a model by extracting temporal features. These features could then be used in a model that can exploit them to arrive at stronger predictions. Still, when limited to single timepoints, predictive performance for phenotypic prediction of yield in crops generally increases as the season progresses, with optimal lead times often suggested to range from approximately one month up until several days prior to harvest. Studies on the use of growth data to estimate yields have demonstrated that access to early flight time points can increase the predictive capability of models, so it is critical that further research be conducted to evaluate this approach [83,85].

Although RF and XGB showed slightly higher R^2^ and RMSE values as compared to the RR, LR, and EN models, the variance explained and repeatability were much more similar between the latter three models and ground-truth yield (Fig. 6). In contrast, RF and XGB showed much higher variance partitioned by the ‘Environment’ component and less by the ‘Genotype’ component, leading to much lower repeatability estimates. It is important to keep in mind that the ‘true’ values for yield can never actually be known, as all methods of collecting and estimating grain yield— including the ground-truth method of using a combine harvester—are prone to varying amounts of error [86]. In addition, the ML models were all trained using the ground-truth data in this study, but they all had their own methods of arriving at their estimates. Therefore, the higher ‘Environment’ variance for the RF and XGB models may not necessarily be ‘wrong’, it just indicates that those models are not estimating yield in a way directly comparable with the combine harvester. Stated another way, it is possible that much more of the variance was truly due to the environmental differences than the ground-truth, RR, LR, and EN estimates captured. Despite this, many sorghum breeders may prefer a model that behaves most similarly to their current methodologies and has a high repeatability from the outset than to take a risk with a model that ostensibly captures less genotypic variation in exchange for giving a ‘more correct’ explanation of variance components. Working within the current plant breeding paradigm ensures a higher likelihood that this technology will be trusted and adopted by sorghum improvement researchers. One additional point of concern would be whether dramatically improved efficiency or throughput is worth sacrificing a small amount of accuracy. This remains to be seen, but prior work has demonstrated that high-quality, efficiently gathered phenomics data may be an excellent complement or even outright substitution for genomics-based crop improvement approaches [87,88].

Regarding selections in a breeding program, Spearman's correlations and confusion matrices demonstrated that the rankings of the BLUPs were very similar between the model predictions and the ground-truth data (Figs. 7 and 8). One of the key considerations for any yield predictions to be deployed in a breeding program is to ensure that poor material is properly being eliminated, and superior material is being progressed within the program. In that respect, the RR, LR, and EN models performed quite well. It is reasonable to suggest that the worst errors would be instances where a top performer was targeted for elimination, e.g. was shown to be in the bottom 50% of the material. This did not occur with most of the ML models; in fact, only SVR had one instance of making this critical error. This implies that the model predictions should not lead to any high-yielding lines being erroneously removed from the set of breeding material, although there is still a great deal of room for improvement since the ML predictions were less reliable at identifying the top 10% vs the top 25% or even 50% (Fig. 8). These results are promising, since breeders will understandably desire rates of genetic gain when using the predicted yields that are consistent with what they would observe if they were using the conventional methods.

## 5 Conclusion

The results of this study indicate that key remote sensing-derived features exhibit moderate to strong correlations with grain yield, while many vegetation indices and panicle traits on their own provide only modest predictive power. Machine learning models built using the full set of Canopy Traits or a combination of Canopy and Panicle features can help to address this deficiency as they consistently achieved high R^2^ values in the context of advanced sorghum trials (approximately 0.74–0.76) and outperformed models relying solely on Panicle Traits, which lagged with R^2^ values around 0.28–0.42. In terms of ranking genotypes, models based on regularization methods (RR, EN, and LR) not only showed correlations closely aligned with ground-truth yield data but also demonstrated similar variance partitioning and repeatability. Our findings underscore that no single feature can serve as an independent predictor of yield, thus necessitating the integration of multiple correlated traits into a comprehensive ML framework.

This study further reveals that, despite the common assumption that more complex models like RF and XGB might capture intricate relationships more effectively, simpler models achieved comparable plot-level yield predictions. This presents a compelling advantage for in-field implementation and broader adoption by sorghum breeders. Notably, when only panicle traits were used, the performance gap widened in favor of tree-based methods; however, the overall accuracy remained insufficient for practical breeding applications. The panicle features also did not improve the combined set in this study, which may indicate that extracted canopy features were capturing similar information. Our study also highlights inherent limitations in using nadir-oriented UAS imagery for assessing panicle characteristics, suggesting that supplementary data (e.g. oblique imaging) may be necessary for capturing critical morphological details and make panicle traits predictive on their own. In addition, these findings are from advanced sorghum trials and using single-date data acquisitions, which could mean that addressing these shortcomings results in stronger performance. Overall, these results support the use of canopy features within streamlined predictive models as a robust and efficient approach, while also calling for further research to validate these findings across diverse populations and environmental conditions.

## Data availability statement

The tabular data and code used in this study can be found in the following GitHub repository: https://github.com/AcePugh/Sorghum_Yield_Prediction_2025/

## Funding

This project was funded by 10.13039/100000199USDA project 3096-21000-023-000-D and 3096-21000-024-000-D.

## Author contributions

N. Ace Pugh: Conceptualization, Data Curation, Formal Analysis, Investigation, Methodology, Project Administration, Supervision, Validation, Visualization, Writing – Original Draft, Writing – Review and Editing. Andrew Young: Data Curation, Formal Analysis, Investigation, Validation, Writing – Review and Editing. Matthew Nesbitt: Data Curation, Resources, Writing – Review and Editing. Chad Hayes: Conceptualization, Data Curation, Methodology, Resources, Supervision, Writing – Review and Editing.

## Declaration of competing interest

The authors declare that they have no known competing financial interests or personal relationships that could have appeared to influence the work reported in this paper.
